# Protective effects of a new generation of probiotic *Bacteroides fragilis* against colitis in vivo and in vitro

**DOI:** 10.1038/s41598-023-42481-8

**Published:** 2023-09-22

**Authors:** Qiuyue He, Min Niu, Jiandie Bi, Na Du, Shumin Liu, Kai Yang, Huanqin Li, Jing Yao, Yan Du, Yong Duan

**Affiliations:** 1 Department of Clinical Laboratory, The First Affiliated Hospital of Kunming Medical University, Kunming, 650032 China; 2 Yunnan Key Laboratory of Laboratory Medicine, Kunming, 650032 China; 3 Yunnan Province Clinical Research Center for Laboratory Medicine, Kunming, 650032 China; 4 Department of Clinical Laboratory, The No. 1 Affiliated Hospital of Yunnan University of Chinese Medicine, Kunming, 650032 China; 5 Department of Blood Transfusion, Yan’an Hospital Affiliated to Kunming Medical University, Kunming, 650032 China

**Keywords:** Cancer, Microbiology, Diseases, Health care, Medical research, Molecular medicine

## Abstract

*Bacteroides fragilis,* one of the potential next-generation probiotics, but its protective mechanism is not yet known. We aimed to characterize the anti-inflammatory effect of *B. fragilis*ATCC25285 and to elucidate its mechanism through in vivo and in vitro experiments. An in vitro model of inflammation by induction of colonic cells with TNF-a, and co-cultured with *B. fragilis* to detect cell viability, apoptosis and invasive capacity. Furthermore, critical proteins of the TLR/NF-κB pathway and the inflammatory cytokines were measured. For animal trials, C57BL/6 J male mice were orally administered *B. fragilis* or PBS once daily for 21 days. Colitis was induced by drinking 2.5% DSS from days 0 to 7. The mice were weighed daily and rectal bleeding, stool condition and blood in the stool were recorded. We found that *B. fragilis* treatment alone was harmless and had no effect on cell viability or apoptosis. While predictably TNF-α decreased cell viability and increased apoptosis, *B. fragilis* attenuated this deterioration. The NF-κB pathway and inflammatory cytokines IL-6 and IL-1β activated by TNF-α were also blocked by *B. fragilis*. Notably, the metabolic supernatant of *B. fragilis* also has an anti-inflammatory effect. Animal studies showed that live *B. fragilis* rather than dead strain ameliorated DSS-induced colitis, as evidenced by weight loss, shortened colon length and enhanced barrier function. The colonic tissue levels of inflammatory cytokines (TNF-α, IL-1β, IL-6) were decreased and IL-10 was increased as a result of *B. fragilis* administration. In conclusion, *B. fragilis* ATCC25285 exhibited anti-inflammatory effects whether in vivo or in vitro, and it may be a potential probiotic agent for improving colitis.

## Introduction

Global incidence and prevalence of inflammatory bowel disease (IBD) has been increasing and has developed into a global scourge over the past 50 years^1^. It is convincing that the development of IBD is the result of a complex interaction between genetic, innate immune and environmental factors. Several studies have confirmed that the gut microbiota plays a key role in the severity and progression of colitis in patients with IBD. Correcting intestinal flora imbalance is an important tool to prevent and treat colitis. Fecal microflora transplantation (FMT) and probiotic administration are currently the main approaches to modify the microbial composition of the gut^2^. In fact, probiotics as a food additive is a more alternative to FMT, which might be more promising for clinical applications due to it's safer, cheaper, and more controllable^3^. However, the lack of clinical effectiveness and unclear molecular mechanisms of intervention render the clinical application of probiotic therapy problematic. Obviously, seeking novel probiotic strains and clarifying response mechanisms will be highly significant.

*Bacteroides fragilis* is a common Gram-negative anaerobic bacterium found mainly in the colon^4^, which is characterized into enterotoxin-producing *Bacteroides fragilis* (ETBF) and non-enterotoxin-producing *B. fragilis* (NTBF) based on the ability to synthesize and secrete *B. fragilis* toxin (BFT)^5,6^. ETBF has been identified as a prevalent opportunistic pathogenic bacterium in clinical infections that promotes chronic inflammation and thus leads to CRC^7–10^. In contrast, NTBF exhibits more probiotic potential and is ranked as a candidate strain for the next generation of most promising probiotics and may be added to the edible list in the future^11^. A widening array of evidence has demonstrated the protection of NTBF in diseases associated with the colon^12^, graft-versus-host disease (GVHD)^13^, bacterial diarrhea^14^ and autism^15^. *B. fragilis* possesses multiple metabolic potentials and various genes linked to polysaccharide utilization have been implicated in influencing host physiology by regulating the composition of the gut microbiota and promoting the production of short-chain fatty acids (SCFAs) in the gut. NTBF have a direct effect on colonic epithelial cells, enhancing barrier effects, reducing membrane permeability, inhibiting colonization by other pathogenic bacteria, and preferentially occupying colonic ecological niches to maintain intestinal flora homeostasis^16^. It also promotes the development of the immune system by secreting polysaccharide A (PSA), which regulates the immune response and maintains the immune homeostasis of the body^17,18^. In addition, NTBF also can alleviate colonic inflammation and inhibit tumorigenesis in the colon, but the exact mechanism is not yet clear^19^.

Our previous work analyzed the gut microflora of patients with colonic adenomatous polyps and found that the abundance of *Bacteroides spp*. was significantly decreased in adenomatous polyp tissue relative to the healthy subjects, in which *B. fragilis* was representative^20^. Takaishi et al.^21^ also observed marked losses in the *B. fragilis* group in the stools of IBD patients. In one recent study, Berberine (BBR) had a modulatory effect on DSS-induced colitis possibly by affecting the growth of *B. fragilis* to regulate intestinal immune cell differentiation. These findings indicated that *B. fragilis* might play a crucial role in protecting against inflammatory bowel injury, which occurs in IBD. However, the precise protective roles *B. fragilis* plays and the underlying mechanisms during the progression of the disease are not completely understood. Therefore, our study aims to investigate the protective effect of *B. fragilis* against colitis and the underlying mechanisms at the cellular and animal levels.

## Results

### *B. fragilis* shows stronger adhesion to LoVo cells than hcoEPIC

Bacteria adhere to the host cell to exert direct ecological effects and physiological actions. The microscopic morphology of *B. fragilis* ATCC25285 was observed after co-culture with cells for 4 h, and the suspension was diluted 10^3^ times and counted, the results indicated that *B. fragilis* adhering to LoVo cells were significantly higher than normal colonic epithelial cells hcoEPIC (*p* < 0.05). However, *B. fragilis* was not detected in the crushed cell suspension after 4 h. Subsequently, the cultivation time was prolonged to 24 h and still failed to detect the invasive bacteria, and the cell morphology was unchanged and the growth status was positive compared to the group with no addition of bacterium. In conclusion, adhesion and invasion assays demonstrated that *B. fragilis* ATCC25285 adhered better to LoVo cells than to hcoEPIC cells, but never showed invasion of both strains (Table 1).

**Table 1:** Comparison of adhesion and invasion ability of *B. fragilis* to hcoEPIC and LoVo cells.

Strain (ATCC25285)	Counts of adherent and invasive bacteria CFU/ml ($\bar{\upchi }$ ± SD)		
	hcoEPIC cell	LoVo cell	*p* value
Adhesion assay(4 h)	(0.86 ± 0.17) × 10^6^	(1.64 ± 0.54) × 10^6^	*p* < 0.05
Invasion assay(24 h)	0	0	*p* > 0.05

Numbers of *B. fragilis* were counted by plating serial dilutions on blood agar plates followed by anaerobic incubation at 37 °C for 24–48 h. All values are mean ± SEM (n = 3).

### Protective effect of *B. fragilis* against TNF-α-induced impairment of cell activity

**Figure 1:**
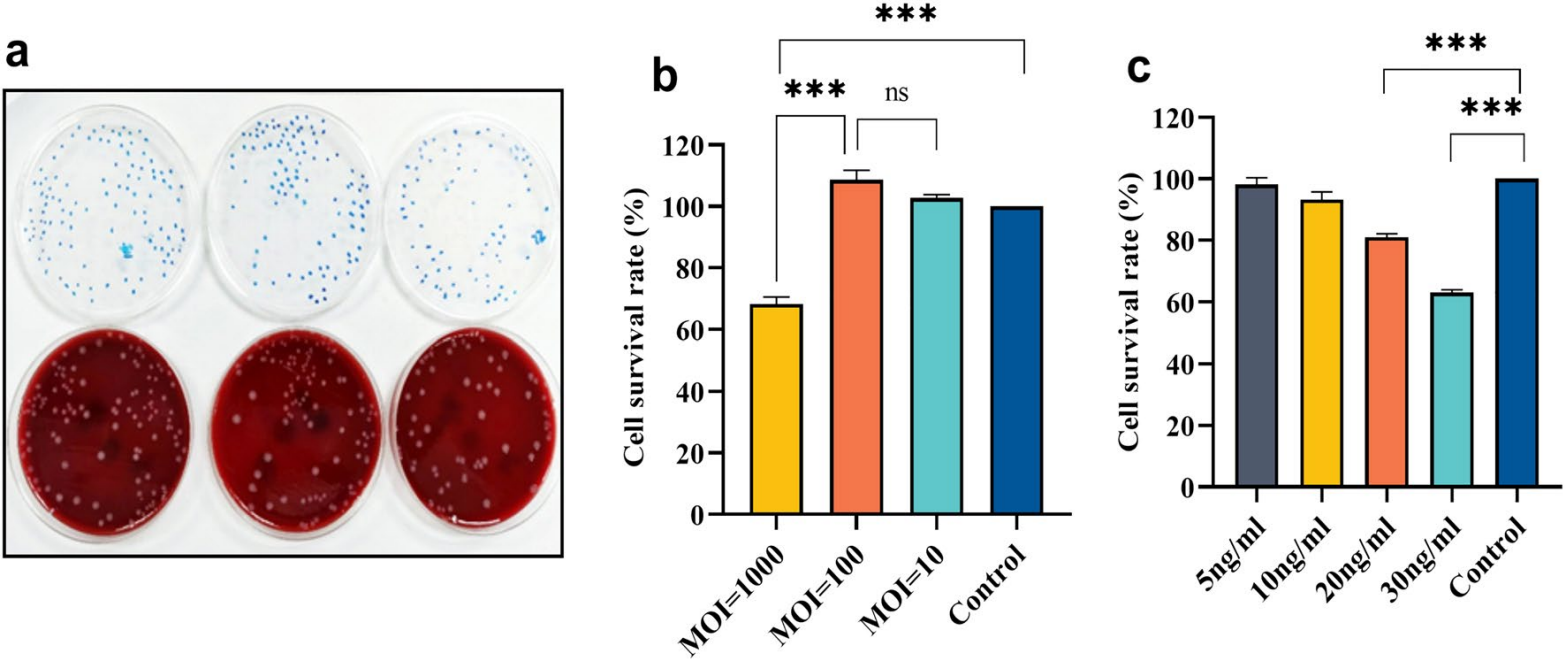
Optimal concentrations of *B. fragilis* and TNF-α were determined. The CCK8 trial was designed to select the optimal concentrations of *B. fragilis* and TNF-α, respectively. (**a**) Results of *B. fragilis* plate counting method(1 × 10^9^ cfu/ml). Cell viability of hcoEPIC cells co-cultured with different concentrations of (**b**) *B. fragilis* and (**c**) TNF-α for 24 h, respectively; ***p < 0.001, ns: not significant. All data show mean ± SEM. CCK-8, Cell Counting Kit-8.

Impacts of *B. fragilis* ATCC25285 and TNF-α on cell motility were initially evaluated. We took *B. fragilis* solution (OD600 = 1), then diluted it 1 × 10^5^ times and counted the colonies on a plate. 103 ± 10.15 colonies were obtained, which was converted to a concentration of approximately 1.03 × 10^9^ CFU/ml (Fig. 1a). Subsequently, various concentrations of *B. fragilis* and cells were co-cultured. We indicated that the *B. fragilis* concentrations of MOI = 100 (108.68 ± 3.0%) and MOI = 10 (98.82 ± 1.4%) had no toxicity to cells (*p* > 0.05), however, MOI = 1000 of *B. fragilis* caused a significant decrease in the survival rate of two strains of cells (68.26 ± 2.3%;* p* < 0.001; Fig. 1b). The cell viability decreased gradually with increasing TNF-α concentration, and 30 ng/ml of TNF-α significantly reduced the viability of cells (63.02 ± 0.9%; *p* < 0.001; Fig. 1c). Therefore, the maximum bacterial concentration with no effect on cells (MOI = 100) and TNF-α concentration (30 ng/ml), the most influential on cell survival, were used as the most optimal experimental condition.

**Figure 2:**
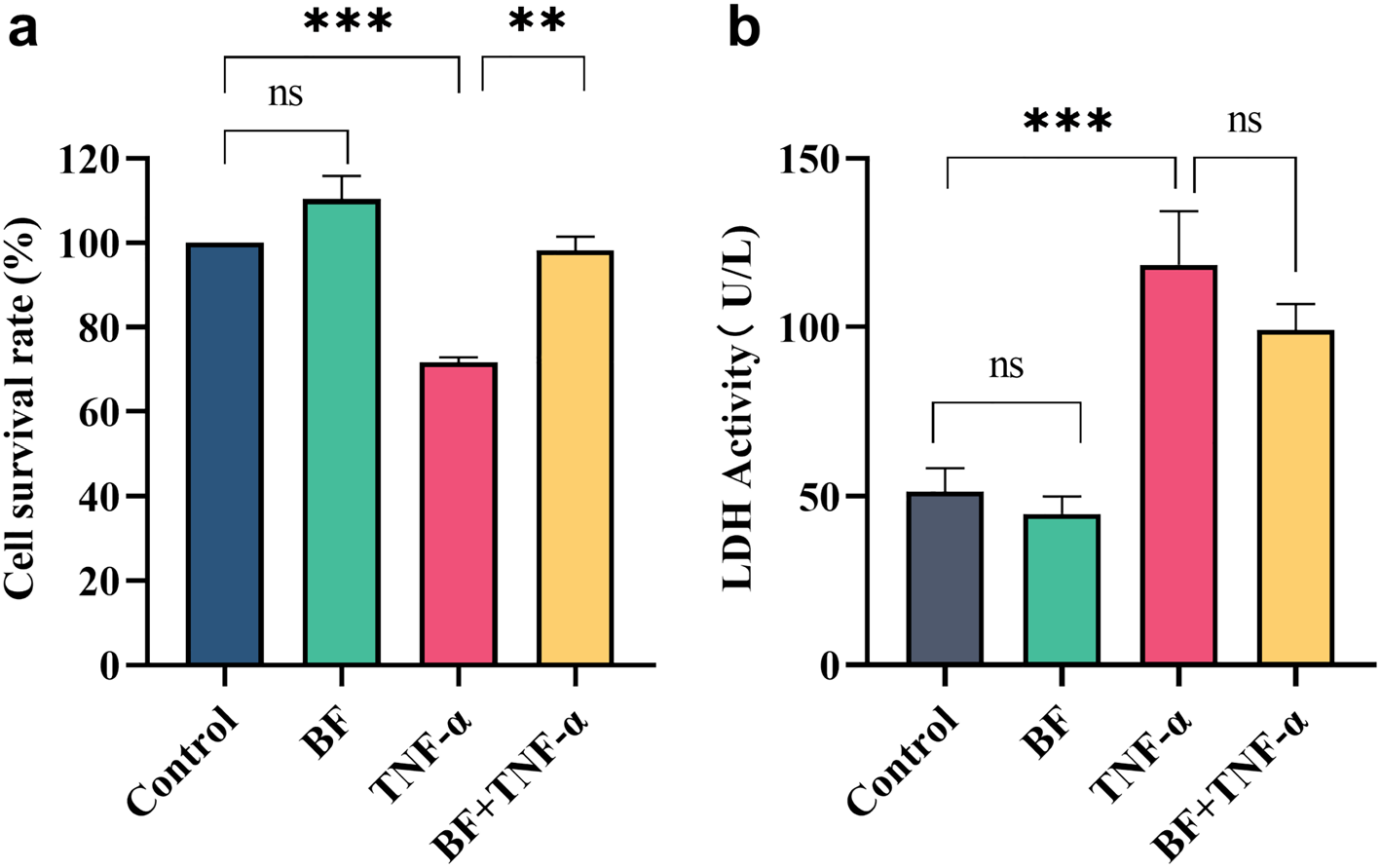
Protective effect of *B. fragilis* against TNF-α-induced impairment of cell activity. (**a**) Cell survival under individual or combined treatment with 1×10^8^ CFU/ml and 30 ng/ml TNF-α. (**b**) Lactate dehydrogenase activity in cell culture supernatants. ***p < 0.001, **p < 0.01, *p < 0.05 ns: not significant. All data show mean ± SEM. *BF* *B. fragilis*, *TNF-α* tumor necrosis factor-α.

To estimate the role of *B. fragilis* ATCC25285 on TNF-α-induced hcoEPIC activity, cell viability and damage were examined by the CCK8 assay and LDH release assay. Treatment of cells with *B. fragilis* only for 24 h did not affect cell survival compared to no addition of the bacteria, indicating that the *B. fragilis* alone did not impair cell activity (*p* < 0.05). Consistently, TNF-α treatment significantly reduced cell survival (71.61 ± 2.96; *p* < 0.001); it was unexpected that *B. fragilis* rescued this injury (98.21 ± 2.95%; *p* < 0.001; Fig. 2a). In addition, LDH was released extracellularly after cell damage or death, therefore, the cell culture supernatants were taken to detect the extracellular LDH release. The results were consistent with the CCK8 assay, and *B. fragilis* decreased extracellular LDH activity compared with TNF-α treatment alone, despite no significant difference in hcoEPIC cells (*p* > 0.05; Fig. 2b). All findings suggested that *B. fragilis* ATCC25285 had a protective effect against TNF-α-induced impairment of cell viability in hcoEPIC cell.

### *B. fragilis* inhibits TNF-α-induced apoptosis in hcoEPIC cells

**Figure 3:**
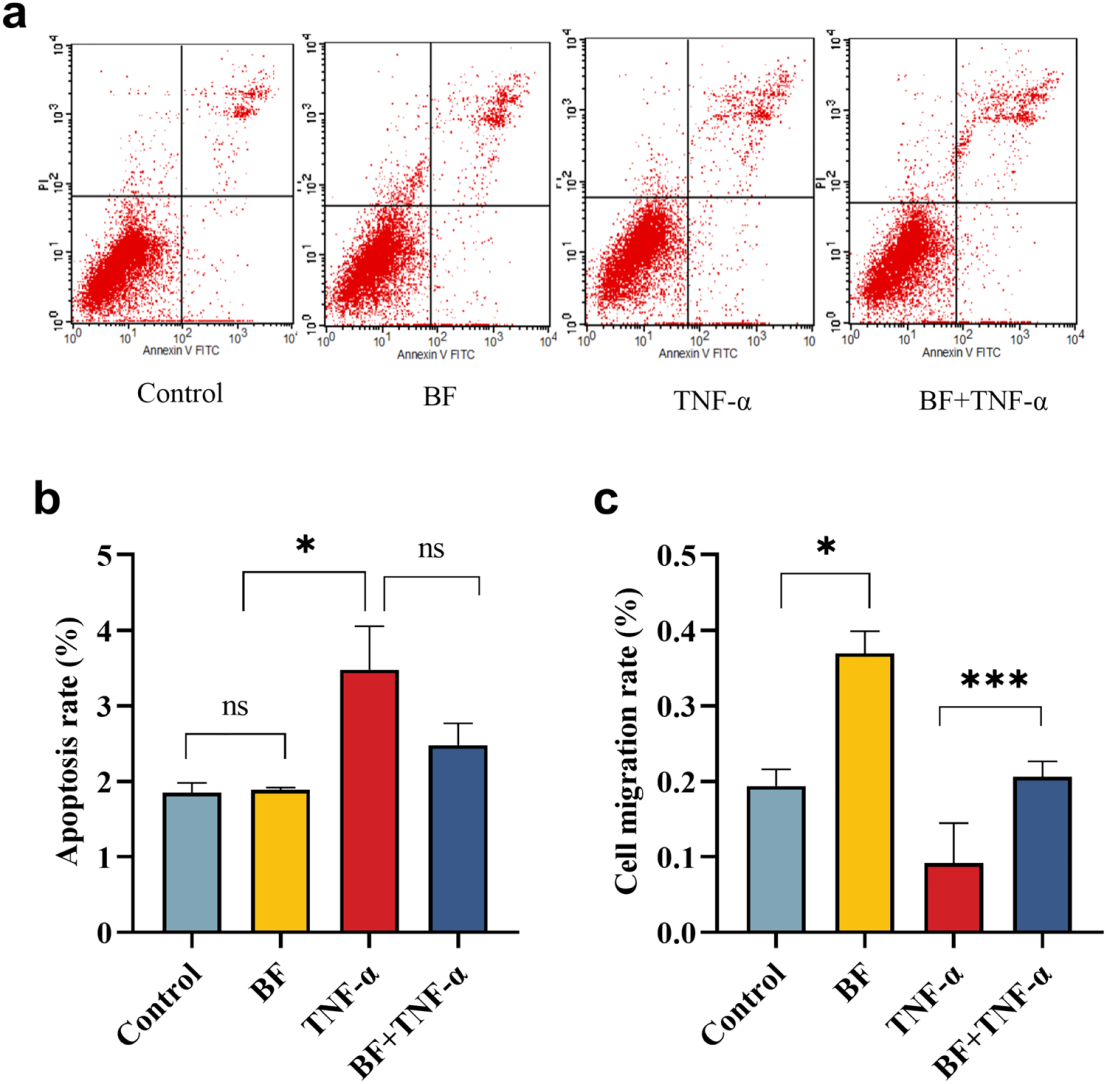
*B. fragilis* inhibited apoptosis and restored migration of hcoEPIC cells induced by TNF-α. Apoptosis rate was valued by flow cytometry in hcoEPIC cells treated with *B. fragilis* and TNF-α alone or together for 24 h. The late apoptotic and early apoptotic cells were located in the Q2 and Q4 quadrant, respectively. (**a**,**b**) apoptosis rate of hcoEPIC cells; (**c**) Migration rate of hcoEPIC cells. ***p < 0.001, **p < 0.01, *p < 0.05 *ns* not significant. All data show mean ± SEM.

Meanwhile, we also examined the influences of *B. fragilis* ATCC25285 on TNF-α-induced apoptosis of hcoEPIC cells. As Fig. 3 shows, the late apoptotic and early apoptotic cells were located in the Q2 and Q4 quadrant, respectively. Analysis revealed no effect of *B. fragilis* treatment alone on apoptosis of cells in hcoEPIC compared to the control group (1.89 ± 0.42%; *p* > 0.05), while TNF-α treatment alone significantly promoted apoptosis (3.48 ± 1.15%; *p* < 0.05). Treatment with *B. fragilis* prior to TNF-α induction led to a reduction of apoptosis in hcoEPIC cells but not statistically significant (2.48 ± 0.50%,* p* > 0.05). The apoptosis assay demonstrated that co-culture of *B. fragilis* ATCC25285 with cells for 24 h had no impact on apoptosis, but tend to reduce TNF- α Induced apoptosis.

### *B. fragilis* restores TNF-α-induced migration of hcoEPIC cells

To explore the role of *B. fragilis* ATCC25285 in the migration of colon epithelial cells in the presence or absence of TNF-α, we evaluated the level of cell scratch healing after 24 h. *B. fragilis* promoted migration of hcoEPIC cells compared to controls (*p* < 0.05). Consistently, 30 ng/ml TNF-α stimulation resulted in inhibition of the migratory capacity of hcoEPIC cell (*p* < 0.001). Notably, *B. fragilis* stimulated the migration of cells compared to TNF-α treatment alone, with a statistically significant increase in the migration of hcoEPIC cells (*p* < 0.001), as shown in Fig. 3c. In conclusion, our data demonstrated that *B. fragilis* ATCC25285 was likely to promote the migration of normal colonic epithelial cells and restore the TNF-α-induced reduction in the migratory capacity of hcoEPIC cells.

### *B. fragilis* playes a protective role in TNF-α-induced inflammation

NF-κB is a widely expressed transcription factor that plays an important role in regulating the immune and inflammatory responses and the programmed cell death. Given the benefits of *B. fragilis* in colonic inflammation, we thus speculated that *B. fragilis* ATCC25285 could regulate the NF-κB pathway. Consequently, we further probed the modulation of the NF-κB pathway by *B. fragilis* in TNF-α-induced inflammatory responses in vitro cellular assay.

**Figure 4:**
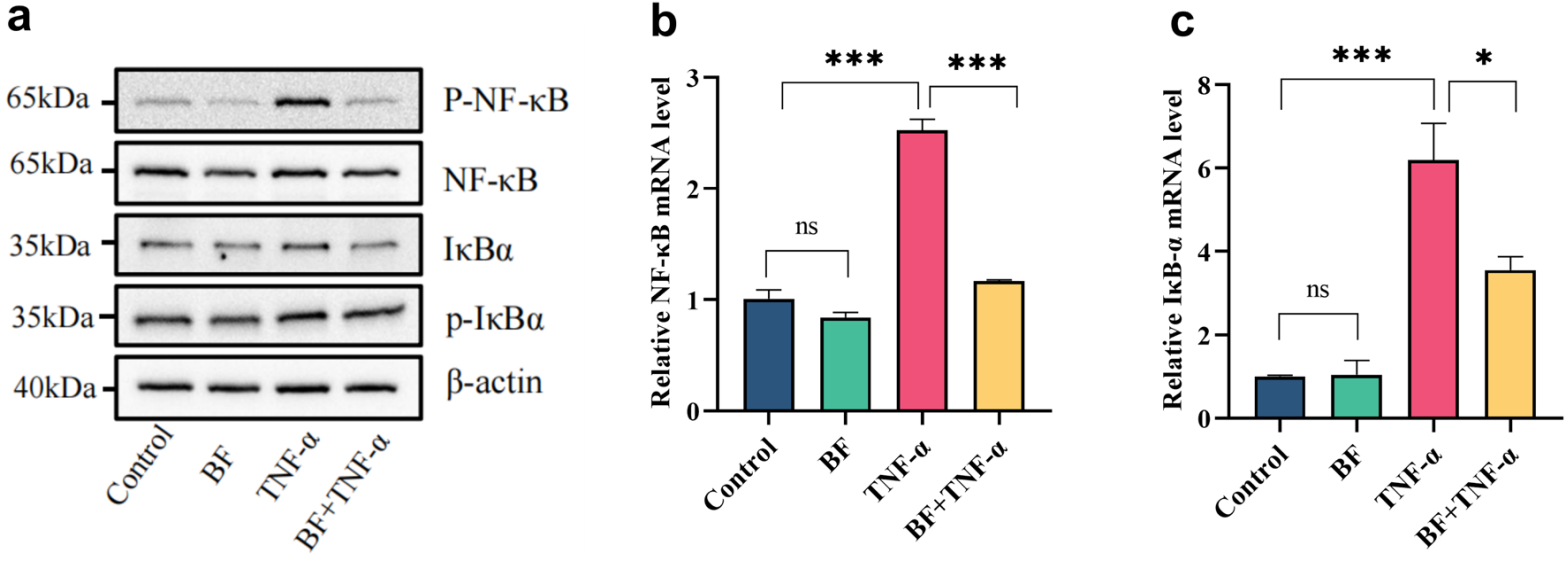
*B. fragilis* inhibits the activation of the NF-κB inflammatory pathway induced by TNF-α. (**a**) Western blot analysis showed that *B. fragilis* reduced the high expression of NF-κB pathways related proteins p-p65, t-p65, p-IκBα, t-IκBα induced by TNF-a, β-Actin was used as an indicator of protein loading. Relative NF-κB mRNA (**b**) and IκB-α mRNA (**c**) level were valued by RT-qPCR assay. ***p < 0.001, **p < 0.01, *p < 0.05 *ns* not significant. All data show mean ± SEM. The original western blot image was visible in Supplementary Material 2.

The expression of NF-κB p65, P-NF-κB p65, IκBα, and P-IκBα, key proteins in the NF-κB pathway, were next analyzed in the context of TNF-α induction. The results showed that TNF-α stimulation alone markedly increased the expression of P-NF-κB p65 and P-IκBα compared to the control group, indicating that TNF-α activated the NF-κB signal (*p* < 0.05), as shown in Fig. 4. Surprisingly, *B. fragilis* down-regulated the expression of P-NF-κB p65 and P-IκBα induced by TNF-α, indicating that *B. fragilis* ATCC25285inhibited the activation of NF-κB signalling induced by TNF-α.

**Figure 5:**
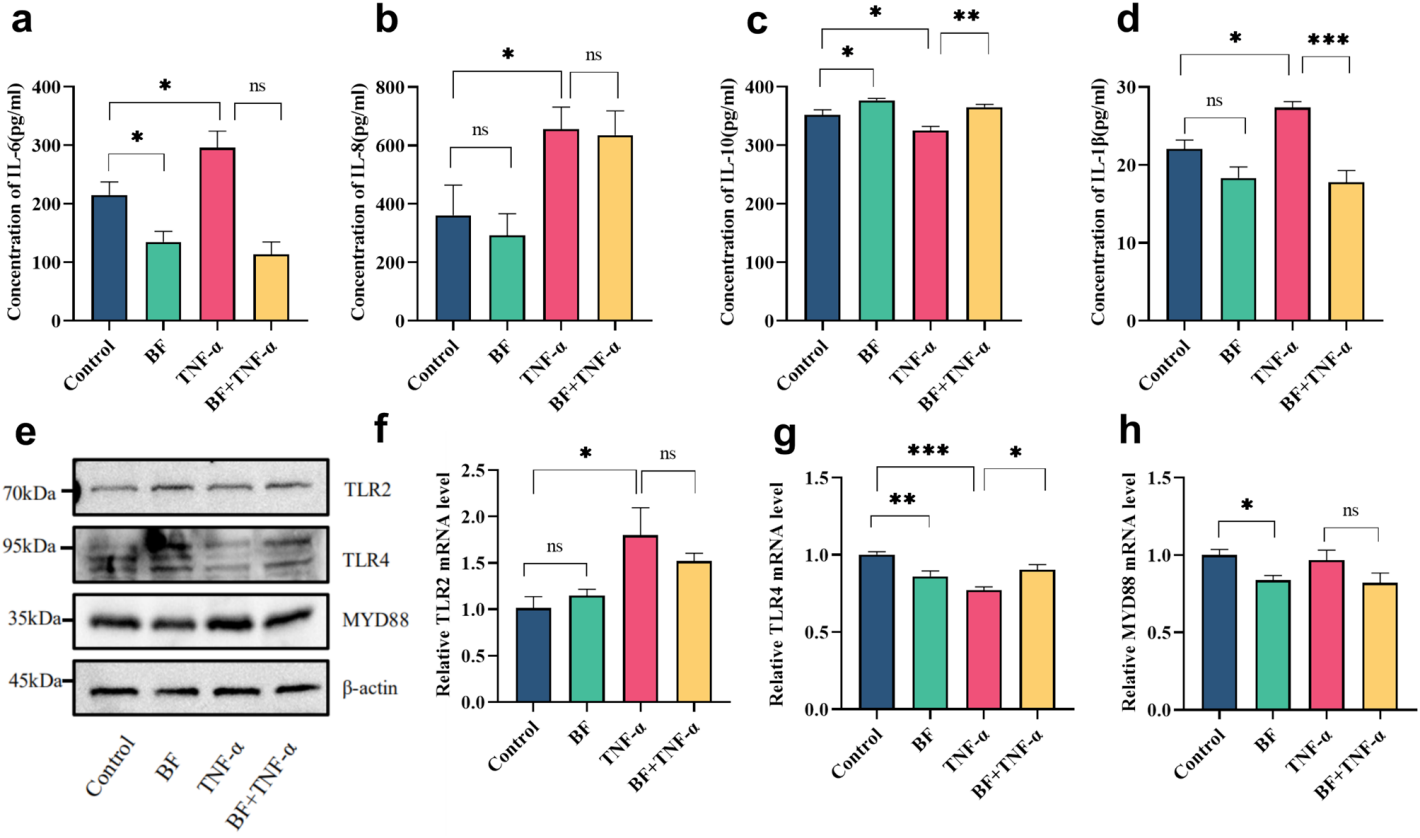
*B. fragilis* inhibited TNF-α-induced generation of inflammation-associated cytokines. Culture supernatant was collected to value the concentrations of cytokines (**a**) IL-6; (**b**) IL-8; (**c**) IL-10; (**d**) IL-1β by Enzyme-linked immunosorbent assay. Expression of TLR2, TLR4, MYD88 were valued by western blot (**e**) and RT-qPCR (**f**–**h**) from molecular and protein level. ***p < 0.001, **p < 0.01, *p < 0.05 *ns* not significant. All data show mean ± SEM. The original western blot image was visible in Supplementary Material 2.

To determine the potential protective effect of *B. fragilis* ATCC25285 against TNF-α-induced inflammation, concentrations of the NF-κB pathway-related cytokines IL-6, IL-8, IL-10, IL-1β were measured. Treatment of cells with *B. fragilis* for 24 h only mildly inhibited the expression of pro-inflammatory cytokines IL-6, IL-8, IL-1βcompared to the control group, with no statistically significant differences except for a decrease in IL-6 in hcoEPIC cells (*p* < 0.05). In contrast, *B. fragilis* gently facilitated the production of IL-10, a cytokine that inhibits inflammation, with a statistically significant increase in IL-10 expression in hcoEPIC cells (*p* < 0.05). Concurringly, the TNF-α stimulation alone led to a significant increase in the pro-inflammatory cytokines IL-6, IL-8, IL-1β (*p* < 0.05), but the effect was inhibited by *B. fragilis*. Similarly, *B. fragilis* also inhibited the reduction of the anti-inflammatory factor IL-10 in hcoEPIC cells caused by TNF-α (*p* < 0.05). These findings pointed to the ability of *B. fragilis* ATCC25285 to inhibit TNF-α-induced production of the pro-inflammatory cytokines IL-1β (*p* < 0.001) and to enhance the generation of the anti-inflammatory cytokine IL-10 (*p* < 0.01) in hcoEPIC (Fig. 5a–d). In Furthermore, TLRs are natural receptors for microorganisms, and have been shown to be targets of action for *B. fragilis.* However, whether *B. fragilis* affects TNF-α-activated inflammation through TLR receptors is not known. Therefore, we examined the expression of TLR2, TLR4 and the downstream protein MYD88D in hcoEPIC cells treated with *B. fragilis* ATCC25285. As shown in Fig. 5e–h *B. fragilis* treatment alone mildly up-regulated TLR2 protein (*p* > 0.05) and down-regulated the expression of TLR4 and MYD88 (*p* < 0.05) in epithelial cells as compared to control. But, effect of *B. fragilis* ATCC 25285 on TLR2 and TLR4 expression in TNFa-stimulated hcoEPIC cells are unclear.

### *B. fragilis* culture supernatant attenuates TNF-α-induced cell death and inflammatory response

**Figure 6:**
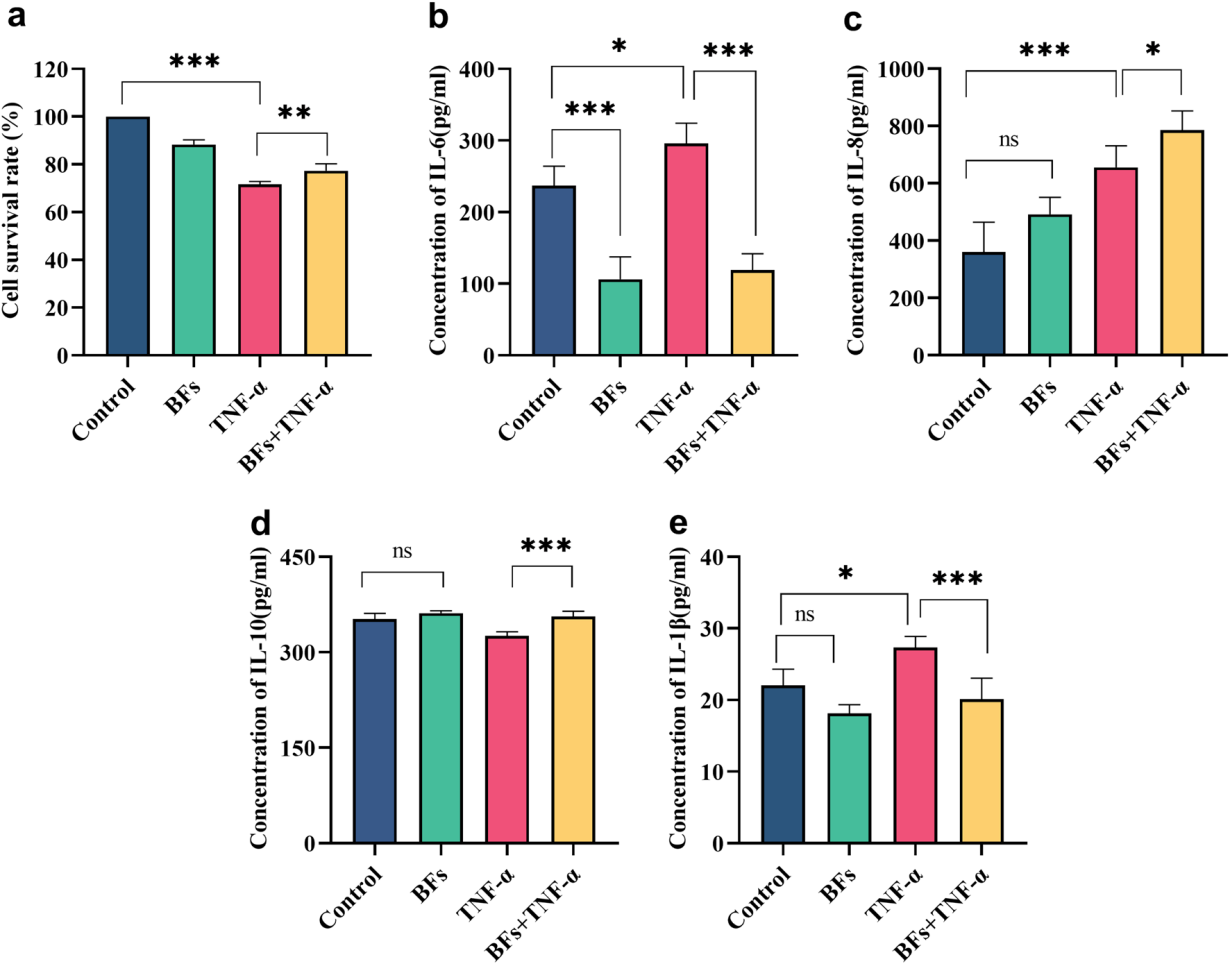
*B. fragilis* culture supernatant attenuates TNF-α-induced cell death and inflammatory response. (**a**) Cell of survival rate of hcoEPIC cells. Culture supernatant was collected to value the concentrations of cytokines (**b**) IL-6; (**c**) IL-8; (**d**) IL-10; (**e**) IL-1β by Enzyme-linked immunosorbent assay. ***p < 0.001, **p < 0.01, *p < 0.05 *ns* not significant. All data show mean ± SEM. BFS, *B. fragilis* supernatant.

To further determine whether *B. fragilis* ATCC25285 metabolites were also involved in protective efficacy, we extracted culture supernatants of *B. fragilis* and analyzed the supernatants for TNF-α-induced cellular damage and inflammatory effects. As illustrated in Fig. 6, the findings showed that *B. fragilis* culture supernatant similarly rescued the TNF-α-induced cell activity from decreasing (*p* < 0.01, Fig. 6a). We also assessed alterations in inflammatory factors and demonstrated that *B. fragilis* ATCC25285 supernatant inhibited TNF-α-induced high expression of IL-1β and IL-6 in hcoEPIC cells, and furthermore that it enhanced the production of the anti-inflammatory factor IL-10, even though no statistical difference was observed. Yet significantly promoted IL-10 expression in hcoEPIC cells relative to TNF-a treatment (*p* < 0.001, Fig. 6b–e). Unlike the *B. fragilis* treatment, the supernatant facilitated IL-8 expression in cells (*p* < 0.05) and displayed a superimposed effect with TNF-a, showing that the *B. fragilis* ATCC25285 supernatant led to higher IL-8 expression in the cells relative to TNF-α effect alone (*p* < 0.05).

### *B. fragilis* alleviates the phenotype of DSS-induced acute colitis

**Figure 7:**
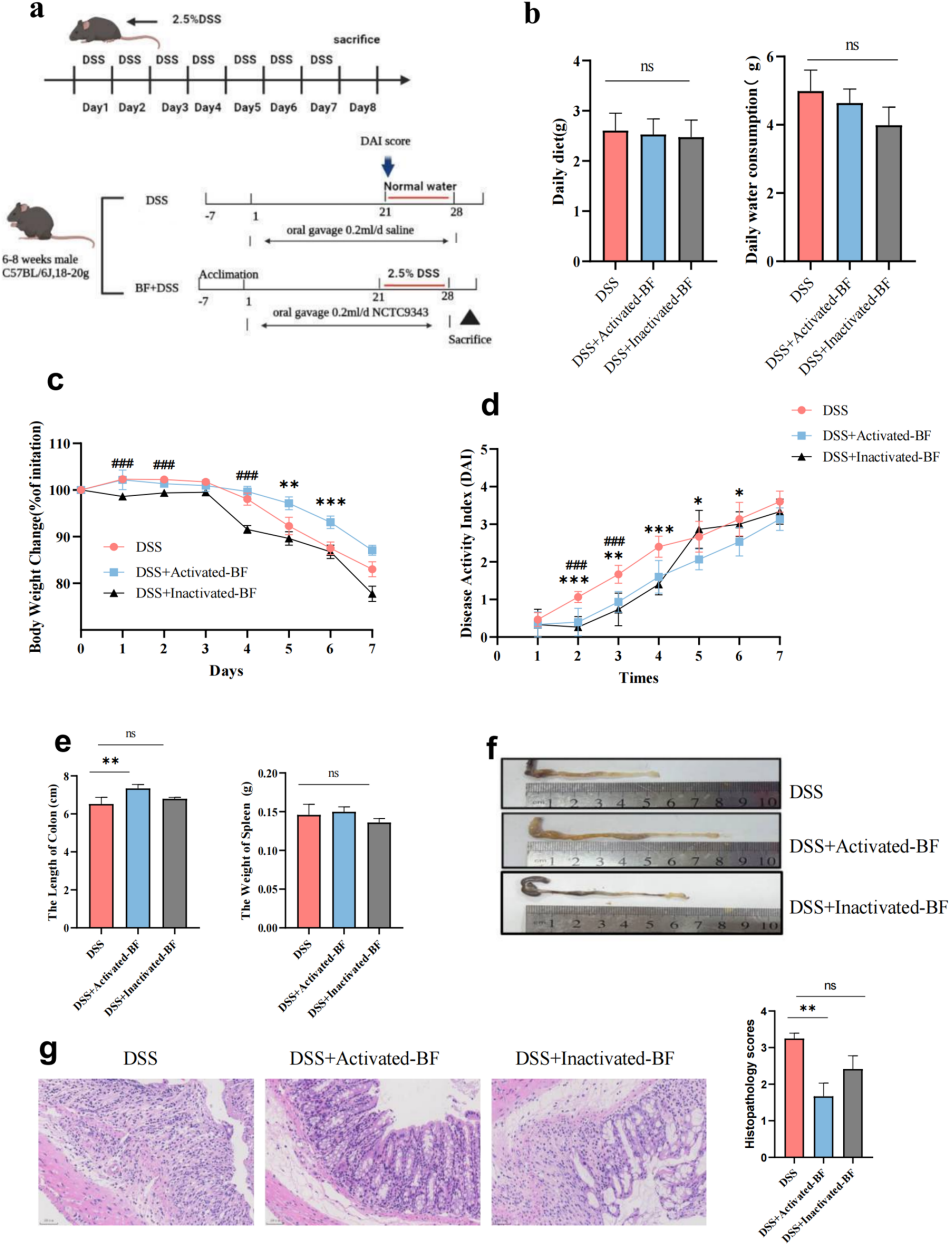
*B. fragilis* alleviated DSS-induced colon injury. (**a**) Scheme of the animal experimental design. Mice were assigned to three groups randomly (DSS, n = 5; DSS + Activated-BF, n = 5, DSS + Inactivated-BF). Mice were continuously administered *B. fragilis* or PBS for 21 days. Colitis was induced by drinking 2.5% DSS from days 0 to 7. On day 8, mice were sacrificed. (**b**) Daily water intake diet for mice. (**c**) The percentage of mouse weight change from days 0 to7day. (**d**) The disease activity index (DAI) scores calculated with time. (**e**,**f**) The colon length and weight of the spleen after DSS and *B. fragilis* administration (left), and representative colons of groups (right). (**g**) H&E and score data. Data are shown as the mean ± SEM. ***p < 0.001, **p < 0.01, *p < 0.05.

To assess the anti-inflammatory effects of *B. fragilis* ATCC25285 in vivo, we established a murine model of acute colitis using DSS induction by feeding mice with phosphate-buffered saline (PBS) or *B. fragilis* ATCC25285 (live and hyperbaric inactivated bacteria) for 21 days, followed by 7 days of colitis induction with 2.5% DSS in drinking water freely (Fig. 7a). Body weight, food intake, water consumption and diarrhoeal scores of mice were measured every other day to exclude any differences in daily diet and water intake. We observed no difference in daily dietary intake among the three groups (Fig. 7b), which excludes any error due to DSS consumption. The results showed mice fed activated-*B. fragilis* showed less weight loss (Fig. 7c) and lower DAI scores (Fig. 7d) than the PBS control group,while the inactivated-*B. fragilis* did not have a similar effect. The extent of blood in the stool was significantly alleviated in mice in the activated-BF + DSS group throughout the experimental period. Moreover, the activated-BF + DSS group showed a trend towards lower intestinal inflammation (colon length) and the spleen weight was similar but not statistically.

Distinct compared to the DSS group (Fig. 7e and f). Pathological staining indicated that the colonic tissues of mice in the DSS group showed local tissue ulceration, absence of mucosal epithelial and intestinal glandular structures, submucosal oedema, severe infiltration of mucosa and submucosa, and even inflammatory cells, leading to higher histological scores (Fig. 7g). Mice in the activated-DSS + BF group had improved histological damage compared to the DSS group, showing less mucosal damage as well as submucosal inflammatory cell infiltration, leading to significantly lower histological scores, but the effect of inactivated-*B. fragilis* was not as effective as that of activated-*B. fragilis*. These results suggested that activated-*B. fragilis* ATCC25285 attenuated the clinical symptoms of DSS-induced colitis in mice rather than inactivated-*B. fragilis*.

### *B. fragilis* enhances intestinal tight junction proteins and exhibits intestinal anti-inflammatory effects

**Figure 8:**
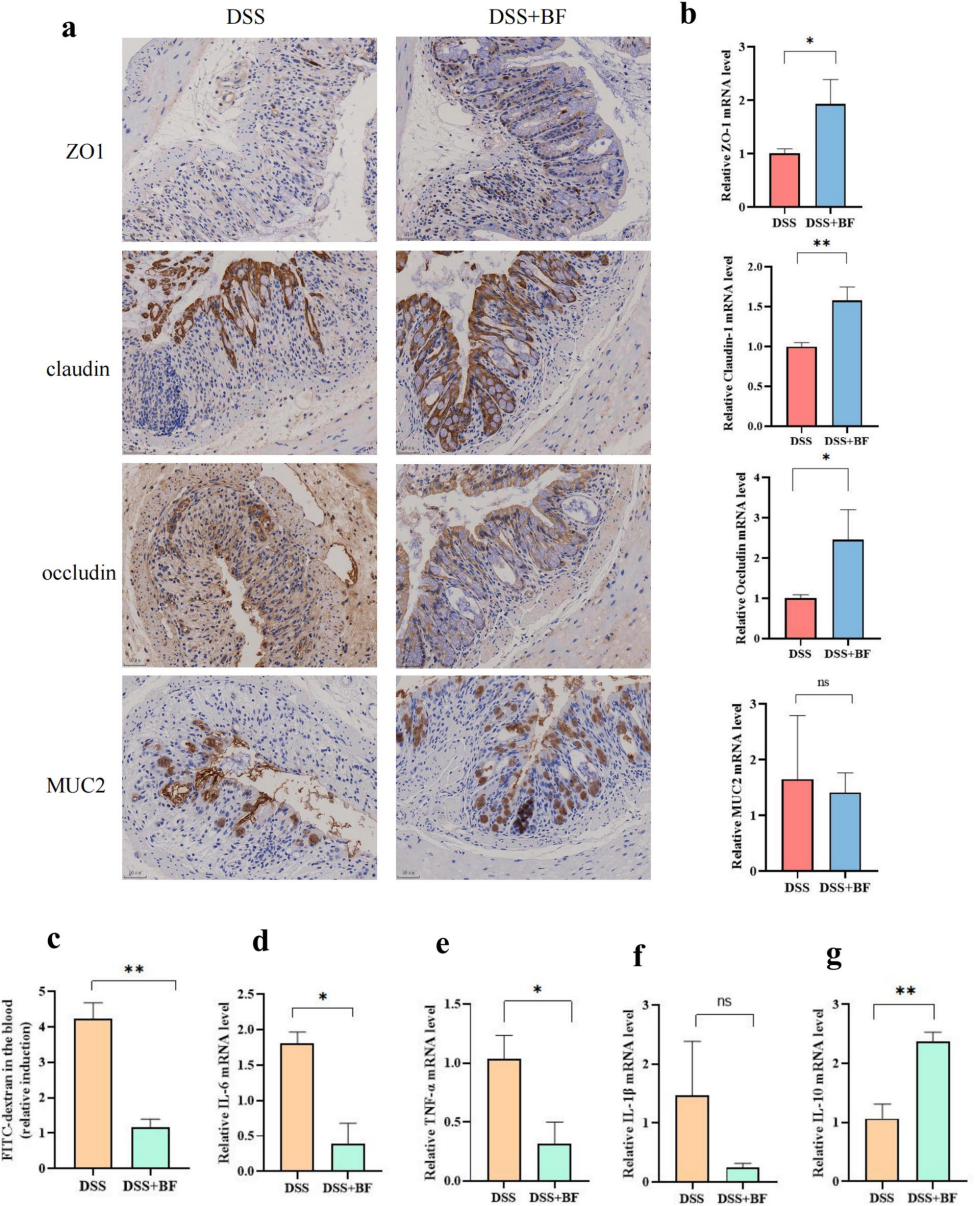
*B. fragilis* enhances intestinal tight junction proteins and exhibited intestinal anti-inflammatory effects. (**a**) IHC staining of the colon (scale bar was 50 μm). (**b**) The concentration of ZO-1, claudin1, occluding, MUC2 in the colon. (**c**) Intestinal permeability measured by determining the plasma concentration of FITC-dextran. (**d**–**g**) Colonic cytokine expression of IL-6, TNF-α, IL-1β, and IL10. (**e**,**f**) Intestinal tight junction protein expression. Data are shown as the mean ± SEM. ***p < 0.001, **p < 0.01, *p < 0.05.

The gut mucosal barrier is the first barrier against the hostile environment and has a key role in the development of ulcerative colitis, mainly formed by the epithelial junction (TJ). The tight junctions are composed of transmembrane proteins (ocludins and claudins) and auxin (ZO), which prevent the spread of pathogens and harmful antigens within the epithelium. The expression of tight junction proteins (ZO1, Claudin, Occludin, MUC2) were analyzed in the colonic tissue sections, and the IHC results showed that the colon tissue in the DSS group presented increased TJ structure defects with destroyed crypts and a disrupted apicalregion, the mean optical density of the proteins was shallow, and the protein distribution was diffuse and discontinuous in DSS group, indicating a low expression (Fig. 8a). Moreover, the levels of the TJ protein expression were remarkably increased in the *B. fragilis* ATCC25285 treatments group.

On the other hand, we have also determined the mRNA expression of these tight junction proteins. We found that *B. fragilis* ATCC25285 treatment significantly increased the expression of ZO1 (*p* = 0.024), Occludin (*p* = 0.027), and Claudin-1 (*p* = 0.009) relative to the DSS group (Fig. 8b).Then, intestinal permeability measured by determining the plasma concentration of FITC-dextran. Notably, FITC-dextran analysis showed a decreased intestinal permeability in mice of the *B. fragilis* ATCC25285 group compared to the DSS group, suggesting an increased barrier function following *B. fragilis* treatment (*p* = 0.001, Fig. 8c) then evaluated alterations in inflammatory factors and showed that *B. fragilis* significantly declined the inflammatory factors IL-6 (*p* = 0.045) and TNF-α (*p* = 0.012) relative to DSS treatment and that expression of the anti-inflammatory factor IL-10 (*p* = 0.001) was increased (Fig. 8d–g).

## Discussion

Our findings showed that *B. fragilis* ATCC25285 reduced TNF-α-induced colonic cell injury, promoted colonic epithelial cell migration in vitro, and preliminarily demonstrated that *B. fragilis* might fight against TNF-α-induced inflammation by down-regulating NF-κB pathway, reducing the release of pro-inflammatory factors IL-6, IL-1β and increasing the production of anti-inflammatory factor IL-10. Notably, we also found the culture supernatant of *B. fragilis* ATCC25285 to be equally anti-inflammatory, as shown in Fig. 6. To clarify the anti-inflammatory effects of *B. fragilis*, we intervened in DSS-induced colitis mice with live *B. fragilis* and a hyperbaric-inactivated dead strain, and the results indicated that live *B. fragilis*, but not the inactivated bacteria, alleviated colitis in mice. Our study elucidates the anti-inflammatory effects of *B. fragilis* ATCC25285 from different models in vivo and in vitro, and notes that *B. fragilis* ATCC25285 metabolites may be key to exerting anti-inflammatory effects.

*B. fragilis* constitutes only 1% of the human intestinal flora but has a profound impact on host physiology^22,23^. Recently, NTBF has been shown to exert beneficial effects on host health by promoting immune system maturation, suppressing abnormal inflammation, and altering the structure of the intestinal flora^24–26^. We revealed that *B. fragilis* ATCC25285 could adhere to tumor cells LoVo and normal colonic epithelial cells hcoEPIC, but the adhesion to tumor cells was significantly higher than that to normal colonic epithelial cells, indicating that *B. fragilis* ATCC25285 has different adhesion abilities to different colonic cells, but the specific adhesion selection mechanism is not clear. This adhesion ability may be associated with its abundant hair structure and the ability of the bacterium to secrete some adhesion molecules such as laminin^27^. Several reports indicated that adherence of *B. fragilis* to colonic cells promotes mucin expression and enhances intestinal epithelial barrier function, while the secreted bacteriostatic substances form a chemical barrier that further strengthens the cellular barrier function^4^. *B. fragilis* could also accumulate on the surface of colonic cells through adhesion, forming a protective biofilm. Han et al.^28^ identified "smectite", an ion-exchange microstructure that preferentially promotes biofilm formation on the surface of probiotic bacteria inside and outside the body. The smectite carrying probiotic biofilm was able to activate dendritic cells via TLR2 signaling in a mouse model and inhibit tumor growth. However, the invasion assay revealed that *B. fragilis* ATCC25285 did not exhibit invasive properties in the two cell strains, and no alteration in cell morphology and activity occurred during the co-culture of bacteria and cells. These outcomes initially suggest that *B. fragilis* ATCC25285 is not harmful to the cells.

TNF-α takes an important role in the development of inflammation^29^. Therefore, we chose TNF-α stimulation to establish an in vitro cellular inflammation model. Results suggested TNF-α led to lower cell viability, higher LDH release and apoptosis, and a significant wrinkling and rupture of the microscopic morphology. However, *B. fragilis* ATCC25285 inhibited the cell deaths and damage caused by TNF-α. Similar to the study found that *B. fragilis* inhibited HT29 apoptosis in intestinal epithelial-like cells^24^. In another study, *B. fragilis* ZY-312 was proven to inhibit apoptosis-induced cell death by downregulating apoptosis-associated proteins^30,31^. In addition, *B. fragilis* was able to restore the functional integrity of the intestinal barrier^15^. Thus, the effect of *B. fragilis* ATCC25285 on TNF-α-induced cell migration was also evaluated in this study. We identified that TNF-α inhibited the migration of hcoEPIC cells, probably due to a significant inhibition of cellular activity at a TNF-α concentration of 30 ng/ml, leading to a decrease in migration rate. This is similar to the studies of Petito et al.^32^ found that TNF-α inhibited the migratory ability of IEC-6 and Caco2 cells. In contrast, *B. fragilis* ATCC25285 treatment alone significantly increased the migration of normal colonic epithelial cells hcoEPIC. The promotion of colonic epithelial cell migration by *B. fragilis* ATCC25285 is most likely a key factor in being able to restore the intestinal mucosal barrier. However, the pre-colonization of *B. fragilis* ATCC25285 accelerated TNF-α-induced cell migration. This may be related to the protective effect of it by inhibiting TNF-α-induced cell injury.

NF-κB is a ubiquitously expressed transcription factor that acts as a critical regulator of immune and inflammatory response and programmed cell death^33^. Our results showed that the NF-κB signaling pathway was slightly activated and it was not statistically significant when treatment with *B. fragilis* ATCC25285 alone. However, the high expression of phosphorylated NF-κB P65 and IκBα caused by TNF-α were significantly down-regulated, indicating that *B. fragilis* could inhibit the activation of NF-κB signaling pathway induced by TNF-α. The protective effect of *B. fragilis* ATCC25285 is largely in a PSA dependent manner and there is evidence that NTBF lacking PSA basically loses its protective activity^18^. *B. fragilis* may secrete beneficial metabolites, such as N-3 polyunsaturated fatty acids and short chain fatty acids to regulate NF-κB signaling pathway, thus exerting anti-inflammatory and tumor suppressor activities^34,35^. Butyrate is the major short chain fatty acid and an important source of energy for epithelial cells, which could regulate cell differentiation and apoptosis, inhibiting intestinal inflammation and the development of CRC^36^. Yin et al.^37^ reported that pre-treatment of HT-29, SW480 and SW620 cells with butyric acid could inhibit TNF-α-mediated nuclear translocation of NF-κB p65 and NF-κB p50 and degradation of IκBα, which is possibly due to the inhibition of deacetylase capacity. In addition, butyrate down-regulated NF-κB signaling pathway to reduce inflammation in rats colitis experiment and to reduce the expression of pro-inflammatory cytokines in intestinal biopsies from patients with Crohn's disease^38,39^. It also reduced the neuritis effect in Alzheimer's disease by inhibition of NF-κB pathway^40^.

We also examined the effect of *B. fragilis* ATCC25285 on the expression of TNF-α-induced inflammatory factors. Results showed the expression of TNF-α-induced IL-6, IL-1β and other pro-inflammatory factors was suppressed by *B. fragilis*. IL-10 is an anti-inflammatory cytokine, IL-10 knockout mice could spontaneously develop colonic inflammation, which is accompanied by increased intestinal permeability and various pro-inflammatory cytokines^41,42^. This study found that *B. fragilis* could promote the secretion of IL-10 in hcoEPIC cells. The possible reason is that the PSA secreted by *B. fragilis* induces the expression of IL-10 by regulating the immune response, thereby inhibiting inflammation. Consistent with this, Mazmanian^43^ and Round^44^ et al. found that *B. fragilis* could protect animals from Helicobacter pylori infection and reduce colonic inflammation by activating regulatory T cells and promoting the secretion of IL-10 to effectively inhibit the production of the pro-inflammatory factor IL-17. Thus, the above results suggest that the protective effect of *B. fragilis* in inflammation is related to the secretion of IL-10. Promisingly, the culture supernatant of *B. fragilis* appears to have similar effects, suppressing high expression of IL-1β and IL-6 induced by TNF-α and increasing the anti-inflammatory factor IL-10, whereas conversely promoting and superimposing the expression of IL-8 induced by TNF-a, indicating that the mechanisms by which the anti-inflammatory effects are exerted by *B. fragilis* itself and the metabolites might be distinct. A recent study by Bernhard^45^ investigated the composition of 10 strains of probiotic bacteria (OMNi bio®AAD10) and the effect of their supernatant after cultivation. They have shown that the supernatant of these bacteria had excellent antibacterial and antifungal effects and promoted the growth of different bacteria in vivo. As yet it remains unclear whether the probiotic effect is triggered by the bacteria themselves or by bacterial metabolites/components such as short-chain fatty acids (SCFA), secondary bile acids or molecular patterns associated with microorganisms e.g. lipopolysaccharides or peptidoglycans. This problem will drive the field of postbiotic research, but our research has not clarified which metabolites act and the mechanisms of action, and further research is needed.

The TLRs family was essential in inflammatory response by recognizing conserved pathogen-associated molecular patterns (PAMPs). TLR2 and TLR4, as important members of this family, have been reported that they are related to the anti-inflammatory effect of *B. fragilis*, but the specific mechanism is not clearly understood till now^30^. Studies have shown that TLR2 is involved in the protection of PSA against colitis and autoimmune encephalomyelitis^46–48^. TLR4 is an important receptor for innate immune recognition of bacterial LPS and its role remains controversial^49^. *B. fragilis* could induce high expression of TLR4 through OMVs (Outer membrane vesicles, OMVs) and significantly increase the anti-inflammatory cytokines in Caco-2 cell line^19,50^. In this study, we showed that *B. fragilis* ATCC25285 treatment alone mildly up-regulated TLR2 protein (*p* > 0.05) and down-regulated the expression of TLR4 and MYD88 (*p* < 0.05) in epithelial cells as compared to control. But, effect of *B. fragilis* ATCC 25285 on TLR2 and TLR4 expression in TNFa-stimulated hcoEPIC cells are unclear. We speculate that possibly the inflammation induced by B. fragilis in response to TNF-a is not mediated by TLR receptors. TLR receptors may be more relevant to LPS-induced inflammation of bacterial origin.This is similar to Ahmadi^50^ et al.'s finding that when stimulated Caco-2 cells with *B. fragilis* and OMVs, the mRNA level of TLR2 significantly decreased and the mRNA level of TLR4 slightly increased, thus increasing the expression of anti-inflammatory cytokines. Besides that, one study reported that PSA secreted by *B. fragilis* could induce Treg cells to express IL-10 and reduce inflammation response in a NOD2 and ATG16L1 dependent manner^41^. However, another studie also showed that *B. fragilis* played a protective role in viral infection through TLR4-induced IFN-β secretion, which was independent of TLR2^26^. Lee^19^ et al. also reported that the TLR4 receptor mediates the inhibitory effect of *B. fragilis* on AOM/DSS-induced colon carcinogenesis in mice. All together, these results suggest that TLR2 and TLR4 may be involved in the anti-inflammatory effect of *B. fragilis*^51^.

In conclusion, our cellular experiments demonstrated that *B. fragilis* ATCC25285 could counteract the TNF-a-induced inflammatory response in normal colonic epithelial cells. Notably, we also found the culture supernatant of *B. fragilis* ATCC25285 to be equally anti-inflammatory. To clarify the anti-inflammatory effects of *B. fragilis* colonic epithelial cells. Notably, we also found the culture supernatant of *B. fragilis* ATCC25285 to be equally anti-inflammat and metabolic supernatants, we obtained dead *B. fragilis* organisms at high pressure and explored the actions of live and dead bacteria body in colonitis in mice. DSS was currently the gold standard in the field of enterocolitis modelling. The advantage of DSS modelling consists in the fact that the symptoms, signs and pathological features of the model animals are identical to those of human ulcerative colitis and it is commonly used as a model for studying the inflammatory mechanisms and anti-inflammatory drugs in human UC. Thus, we performed animal experiments to assess the role of *B. fragilis* ATCC25285 in DSS-induced acute colitis. The results demonstrated that preimplantation of living *B. fragilis* ATCC25285 rather than dead strain was effective in alleviating weight loss and colonic shortening caused by DSS, reducing diarrhoea scores, and increasing the expression of tight junction proteins as well as decreasing inflammatory factors. Zheng^52^ explored the effects of berberine (BBR) on intestinal bacteria and the inflammatory response induced by DSS in mice with colitis. BBR eliminated DSS-induced intestinal flora disturbances in mice, particularly increased *B. fragilis* in vivo and in vitro. *B. fragilis* decreased the interleukin-6 induced by dendritic cells through some heat-resistant component rather than nucleic acids or proteins. These studies demonstrated that high abundance of *B. fragilis* may contribute to the resistance against DSS-induced colitis in mice. A large part of the anti-inflammatory effect of *B. fragilis* may be attributed to active substances in the metabolism of the living bacteria body.

*B. fragilis*-mediated protective mechanism is complex but extracellular vesicles from *B. fragilis* and its outer membrane PSA are also effective in NF-κB-mediated inflammation which is not confined to colon diseases. In addition, *B. fragilis* colonization-induced intestinal metabolites (SCFA) modulates immune cell development (Treg) and intestinal permeability in vivo and in vitro. Our study revealed that *B. fragilis* ATCC25285 inhibited the NF-κB signalling pathway and pro-inflammatory factors in a TNF-α-induced inflammatory cell model, and that this effect does not appear to be mediated by the TLR receptor. However, we did not characterise in this study whether the anti-TNF-induced inflammatory effects of *B. fragilis* are dependent on PSA. Given the importance of PSA, the construction of PSA-deficient *B. fragilis* strains to clarify the anti-inflammatory mechanism is necessary in future studies. It was notable that we found that *B. fragilis* ATCC25285 frontal culture supernatants were equally effective against TNF-a-induced cellular inflammation, and that subsequent gavage of inactivated bacteriophage to DSS-induced colitis mice did not show efficacy. The findings revealed that the active components in the culture supernatant of *B. fragilis* ATCC25285 may be the key to its anti-inflammatory effect, so future studies to isolate the active components of metabolised species of *B. fragilis* and to investigate their functional mechanisms would be of significance.

## Conclusions

In conclusion, we have shown that the anti-inflammatory effect of *B. fragilis* through in vivo and in vitro experiments. Our results supported the *B. fragilis* is a promising probiotic candidate for colitis-related diseases treatment. In the future, mechanism‐based interactions between the host and gut microbiota and human trials will be needed to confirm the long‐term safety and efficacy of *B. fragilis*. Our findings highlight that the *B. fragilis* has potential as a next-generation probiotic strain to assist in the treatment of inflammatory diseases of the colon, and characterization of the probiotic properties at a strain level will offer the opportunity to find novel strategies for microbiome therapeutics.

## Materials and methods

### Bacterial strains and culture conditions

*Bacteroides fragilis* strain ATCC25285 was purchased from American Type Culture Collection (ATCC; Manassas, VA, USA). The strain was cultured in Brain Heart Infusion (BHI) broth anaerobically at 37 °C in anaerobic environment containing 5% H_2_, 10% CO_2_ and 85% N_2_ for 48 h. BHI was supplemented with 0.0005% hemin and 0.5 mg/mL vitamin K1 for optimal growth (BHIS)^53^ *B. fragilis* was centrifuged, washed three times with sterile PBS solution, and then was adjusted to 10^9^ CFU/ml in sterile PBS through measuring the optical density at 600 nm using spectrophotometer (Thermo, USA). The culture supernatant of *B. fragilis* was filtered through a 0.25 μm filter and prepared for usage.

### Co-culture and processing of *B. fragilis* and cells

Human normal colonic epithelial cells hcoEPIC and colonic adenocarcinoma epithelial cells LoVo were kindly donated by Kunming Institute of Zoology, Chinese Academy of Sciences. Two cells were cultured in RPMI 1640 medium with 10% fetal bovine serum (FBS; BI) and 1% penicillin/streptomycin in 5% CO_2_ at 37 °C in humidified atmosphere. Recombinant Human TNF-αlpha (TNF-α) was dissolved in PBS, adjusted to a concentration of 100 μg/ml and stored at − 80 °C. Cells with 80–90% confluence were detached from culture plates by using 0.25% Trypsin–EDTA solution. After 3 to 4 passages, cells were used for different assays. The specific experimental groupings are as follows: (1) Control group; (2) *B. fragilis* group, co-cultured hcoEPIC with *B. fragilis* at a concentration of 1 × 10^8^ CFU/ml (MOI = 100) for 24 h; (3) TNF-α induction group, hcoEPIC cells were only treated with 30 ng/ml TNF-α for 24 h; (4)*B. fragilis* + TNF-α group, the cells were co-cultured with *B. fragilis* for 4 h firstly, and then TNF-α was added to treat the cells for 24 h.

### Animal experiments

All animal experiments were approved by the Animal Care and Use Committee of Kunming Medical University (protocol code kmmu20221532 of 15 July 2022; Yunnan, China) and were performed in accordance with the University's Guide for the Care and Use of Laboratory Animals. Figure 7a is a schematic diagram of the experimental protocol. 6–8-week-old male C57BL/6 J mice purchased from the Department of Zoology, Kunming Medical University. Each group of 5 mice was housed in isolation cages and kept under the following automatically controlled conditions: light (12 h light, 12 h dark), temperature (24 °C) and relative humidity (55%). After 7 days of acclimatization feeding, mice were placed in one of the following groups: (1) DSS-control (DSS), (2) Activated- *B. fragilis* + DSS, (3) Inactivated- *B. fragilis* + DSS. *B. fragilis* including live organisms and high pressure to obtain dead cells of this strain.These mice were administered, respectively, PBS (200µL of suspension per mouse), *B. fragilis* (10^9^ CFU/mL) every other day for 3 weeks. Suspensions were given by oral gavage. To induce experimental acute colitis, mice in the two groups were provided drinking water containing low-molecular-weight DSS (2.5% DSS with a molecular weight of 36,000–50,000 Da, MP Biomedicals, Solon, OH) for an additional week after the 3-wk study period. Daily clinical assessment of DSS-induced colitis was performed, including measuring body weight (BW) and food intake and noting rectal bleeding, stool conditions, and blood in stool using the disease activity index (DAI) scoring system^54^. The DAI score was calculated as the scores of BW loss (0 = none; 1 = 1 to 5%; 2 = 6 to 10%; 3 = 11 to 20%; 4 =  > 20%), stool consistency (0 = normal; 1 = Soft stools;2 = Very soft stools; 3 = Watery stools (Diarrhea), and stool blood (0 = negative; 1 = Positive( +);2 = Positive(+ +);3 = Positive(+ + +); 4 = Visible rectal bleeding, judgment of the degree of positivity followed the instructions provided by the manufacturer. After 4 weeks, all mice were killed for downstream experiments.

### H&E for pathological analysis

The colon tissue was fixed in 10% formalin and then embedded in paraffin. Sections were stained with H&E for pathological analysis. A scale of 0 to 4 was used to describe the severity of inflammation (0 = absent, 1 = mild, 2 = moderate, 3 = severe), the degree of inflammatory involvement (0 = absent, 1 = mucosal, 2 = mucosal and submucosal, 3 = transmural), and the extent of epithelial/cryptal damage (0 = absent, 1 = basal 1/3, 2 = basal 2/3, 3 = loss of crypts, and 4 = destruction of crypts and surface epithelium). Each parameter was calculated and summed to obtain the mean value.

### FITC-dextran intestinal permeability assay

The mice were fasted from water and food for 4 h on the 7th day of 2.5% DSS treatment, then gavaged at 0.6 mg/g (FITC-dextran) per mouse, blood was taken from the eyeballs of the mice after placing them in a dark environment for 4 h, centrifuged for15min at 7500 rpm/min, and supernatant of each sample was taken. Add 50ul of PBS + 50ul of serum to be tested in a black opaque 96-well plate, and detect the OD value with a spectrophotometer (excitation wavelength 490 nm/emission wavelength 530 nm), the higher the content represents, the better intestinal permeability and the intestinal barrier is severely damaged.

### Immunohistochemistry

Paraffin-embedded tissue sections were rehydrated and antigenically repaired using sodium citrate buffer. The sections were then incubated overnight at 4 °C with primary antibodies targeting ZO1, occludin, claudin, and MUC2 (servicebio, China, 1:1000 dilution). The sections were then washed and subsequently incubated with goat anti-rabbit IgG-peroxidase (servicebio, China, 1:1000 dilution) for 30 min at room temperature. After washing, the sections were stained with DAB. Finally, the sections were dehydrated, made transparent in xylene, and fixed with neutral gum.

### Bacterial adhesion assay

hcoEPIC and LoVo cells were seeded at 1 × 10^5^ cells per well in a 12-well tissue culture plate. Cells were washed three times with sterile PBS to remove residual serum and antibodies, antibodie-free RPMI 1640 medium containing 10% FBS was added to each well in equal volume, and *B. fragilis* was co-cultured with cells at a ratio of MOI = 100 at 37 °C in 5% CO_2_ incubator for 4 h. Then the cells were washed three times, 200 μl of 0.5% Triton-X100 was added and the cells were lysed at 37 °C for 15 min. After that, another 800 μl of sterile distilled water was added, mixed and diluted 10^3^ times, a total of 10 μl of bacterial solution was evenly spread on Columbia blood agar plates and incubated at 37 °C for 48 h under anaerobic environment, and then the colony number was counted.

### Invasion assay

hcoEPIC and LoVo cells were co-cultured with *B. fragilis* (MOI = 100) for 24 h as described previously. Subsequently, cells were washed 3 times with PBS, and then 500 μl of RPMI 1640 basal medium comprising 100ug/ml gentamicin was added and incubated in a 5% CO_2_ incubator at 37 °C for 1 h to kill extracellularly attached bacteria. Then, cells were treated with 200 μl of 0.5% Triton-X100 and lysed at 37 °C for 15 min immediately. Neutral distilled water was added to 800 μl and mixed, 10 μl of the stock solution was laid on Columbia blood agar plates and colonies were counted after 48 h.

### CCK 8 assay

Cells in well growth status were seeded into 96-well plates at a density of 1 × 10^4^ per well and incubated at 37 °C in a 5% CO_2_ incubator for 24 h. Later, the cells were treated with various concentration grades of *B. fragilis* (MOI = 1000; MOI = 100; MOI = 10) and TNF-α (5 ng/ml, 10 ng/ml, 20 ng/ml, 30 ng/ml) for 24 h, and the cell viability was detected by CCK8 Reagent Kits (Proteintech, Wuhan, China) to select the most optimum experimental concentration. In addition, the cells were treated with this optimal concentration in the grouping mentioned above, and cell viability was tested measured accordingly. In brief, after cells were treated as indicated, CCK-8 solution was added to the culture medium, and were incubated for another 1 h. Cell activity was measured at 450 nm using a Microplate Reader.

### LDH release assay

Cell damage or demise triggers the release of lactate dehydrogenase (LDH) into the extracellular compartment, and measurement of extraneous LDH activity can help assess cell damage or death. LDH catalyzes the production of pyruvate from lactate and produces an absorption peak at 450 nm. The color shade is proportional to the pyruvate concentration, thus the LDH activity was calculated by measuring the OD value. Specifically, cells were inoculated into 6-well plates at a density of 1 × 10^5^ per well, incubated for 24 h and then treated as described above, followed by the collection of cell culture supernatant for the assay. LDH kit (Elabscience, Wuhan, China) was used to assess cell injury according to the manufacturer’s protocol. Absorbance value was obtained at 450 mm using enzyme-labeled instrument (Thermo, USA).

### Flow cytometric analysis

Annexin V/PI Apoptosis Detection Kit (Keygen, Nanjing, China) was used to assess cell apoptosis. In short, Preparation by seeding hcoEPIC cells at a density of 1 × 10^5^ cells/ml into 6-well plates. 24 h later, cells were treated with *B. fragilis* and TNF-α alone or in combination as described above in terms of grouping. Single cell suspension was prepared and centrifuged at 300 g for 5 min. The suspension was collected, washed and re-suspended in 300 μl of binding buffer containing 5 μl FITC-annexin V and 5 μl propidium iodide (PI). Gently vortexed the cells and incubated in the dark at 25 °C for 15 min. The degree of cell apoptosis was assessed by flow cytometry (BD Biosciences, CA, USA), based on the manufacturer’s instructions.

### Scratch assay

Scratch assay were done as previously reported^55^. Briefly, the cell monolayer formed in the plate was scraped off with the tip of a 100μL sterile pipette and the isolated cells were washed off with PBS. 0 and 24 h latter, wound width was observed and photographed. Wound healing rates of different treatment groups were collected and calculated.

### Quantitative real time polymerase chain reaction (qRT-PCR)

Total RNA from hcoEPIC cells was isolated using total RNA extraction kit (TIANGEN, Beijing, China) according to the manufacturer, and detected the concentration, purity and integrity of RNA. 1 μg RNA was reverse transcripted into cDNA by 5xAll-ln-one RT MasterMix kit (abm, Canada). TLR2, TLR4, MYD88, NF-κB, IκBα, IL-6, IL-10, IL-1β, TNF-α, and ZO1, Occludin, Claudin-1, MUC2 were quantified by using SYBR Green PCR Master Mix (TIANGEN, Beijing, China) on a Light Cycler^®^96 real-time PCR detection system (Roche Molecular Systems, Inc). The reaction conditions were as follows: pre-denaturation at 95 °C for 1 min, followed by 40 cycles of denaturation at 95 °C for 5 s, annealing at 60 °C for 15 s and extension at 72 °C for 20 s. β-actin was included as internal reference and data were analyzed by 2^−△△Ct^ method. The primer sequence of genes was exhibited in additional file1.

### Western blotting

The protein expression was measured by western blotting analysis. Cells with PBS and lysed in ice-RIPA lysis buffer containing protease inhibitors. The supernatant was separated from cell debris using centrifugation at 14,000 rpm for 20 min at 4 °C. The proteins were quantified using bicinchoninic acid (BCA) method, sodium dodecyl sulfate polyacrylamide gel electrophoresis (SDS-PAGE) was used to separate the proteins. The extracted proteins were transferred to polyvinylidene fuoride (PVDF) membranes. After blocking for 2.5 h in 5% (w/v) skimmed dry milk in Tris-buffered saline containing 0.1% Tween-20 (TBST) at room temperature, the membrane were washed and incubated with appropriate primary antibodies at 1:1000 dilution [Abs against TLR2; TLR4; MYD88; NF-κB p65; p-NF-κB p65 ; IκBα; p-IκBα ] and 1:5000 dilution β-actin in 5% (w/v) BSA in TBST and incubated overnight at 4 °C. Then, the membrance were washed three times with TBST and incubated with secondary antibody (goat anti-rabbit IgG, 1:5000) at room temperature for 2 h. The reaction bands were detected using enhanced chemiluminescence (ECL) (GenDepot, Katy, TX, USA) and observed using Chemiluminescence imaging system (GE Healthcare, Beijing, China).

### ELISA assay

Culture supernatant was collected from 6-well plates and the concentrations of cell cytokines (IL-6; IL-8; IL-10; IL-1β) were valued using enzyme-linked immunosorbent assay (ELISA) kits (Proteintech, Wuhan, China) and normalized to cell protein concentrations. Each assay was carried out under protocols supported by the individual manufacturers.

### Statistical analysis

Statistical calculations were performed using SPSS 19.0 software and GraphPad Prism 6.0 software. All the results are shown as mean ± Standard Error of Mean (SEM). Statistical differences between experimental groups were evaluated by Student’s t tests and one-way analysis of variance (ANOVA) followed by post hoc analysis using LSD comparison tests for multiple comparisons. *p* values of 0.05 were considered statistically significant.

### Institutional review board statement

All animal experiments were approved by the Animal Care and Use Committee of Kunming Medical University (protocol code kmmu20221532 of 15 July 2022; Yunnan, China) and were performed in accordance with the University's Guide for the Care and Use of Laboratory Animals.

### Supplementary Information

Supplementary Information 1.

Supplementary Information 2.

## Data Availability

The data used to support the findings of this study are available from the corresponding author upon request.
