# Supplementary material for: Protective effects of a new generation of probiotic Bacteroides fragilis against colitis in vivo and in vitro
Source: Sci Rep. 2023 Sep 22;13:15842. doi: 10.1038/s41598-023-42481-8 (PMC10517118; doi:10.1038/s41598-023-42481-8)
Supplement: Supplementary file 1 — Supplementary Information 1. [file 41598_2023_42481_MOESM1_ESM.pdf]

Supplementary material 1

Table2. Primer sequences used in quantitative real-time polymerase chain reaction (qPCR) Analysis.

Gene name	Forward/Reverse Primer Sequence (5-3)
TLR2	F：AATCCTCCAATCAGGCTTCTCTG
	R：CTTTTTACAGCTTCTGTGAGCCC
TLR4	F：CTTCTCAGCAGGAACACTTACCT
	R：GTACCCACTGTTCCTTCTGGATT
MYD88	F：ACTGTAATGATGTGAGCAGGGAG
	R：GTACAAAGTTGGTGGGAAAGCTC
NF-κB	F：AAGATCTGCCGAGTAAACCG
	R：TCCCGTGAAATACACCTCAA
IκB	F：TGCACTTGGCCATCATCCAT
	R：TCTCGGAGCTCAGGATCACA
β-actin (Human)	F：AGCAGATGTGGATCAGCAAG
	R：TAACAGTCCGCCTAGAAGCA
IL-6	F：TGCCTTCTTGGGACTGAT
	R：TAAGCCTCCGACTTGTGA
IL-1β	F：GAAATGCCACCTTTTGACAGTG
	R：TGGATGCTCTCATCAGGACAG
IL-10	F：CCCAGAAATCAAGGAGCATT
	R：CTCTTCACCTGCTCCACTGC
TNF-α	F：CCCTCACACTCAGATCATCTTCT
	R：GCTACGACGTGGGCTACAG
ZO1	F：GGCCTTGGCCTAGCATACAC
	R：GTCTTCATTTGACCCTCCCTC
Occludin	F：TCACTTTTCCTGCGGTGACTT
	R：GGGAACGTGGCCGATATAAT
Claudin-1	F：AGCTGTGCATGGCCTCTTGT
	R：CCAATGTCAATGGCAACACCC
MUC2	F：TGCTGACGAGTGGTTGGTGAAT
	R：GATGAGGTGGCAGACAGGAGAC
β-actin	F：ATTACCCGCCCGACAATAGG
(Mus)	R：CATGAGTCAGCTAGGCTAGAA

F forward, R reverse

Supplementary material 2

Figure4a

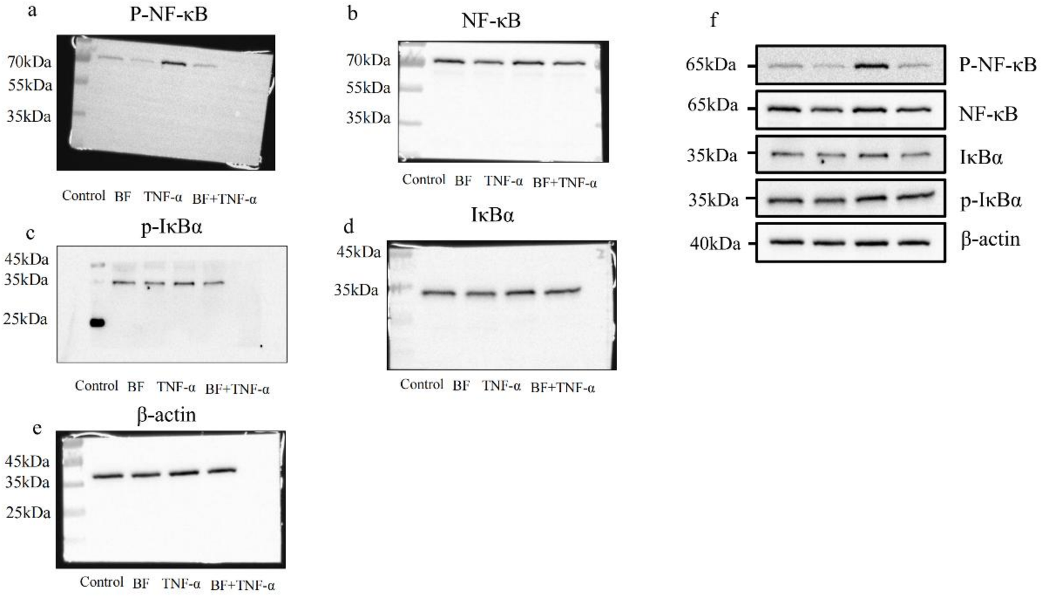

Protein bands in Figure4a. a-e: Original image of p-NF-κB, NF-κB, p-IκBα, IκBα, β-actin.
f: Crop diagram of p-NF-κB, NF-κB, p-IκBα, IκBα, β-actin.

Figure 5e

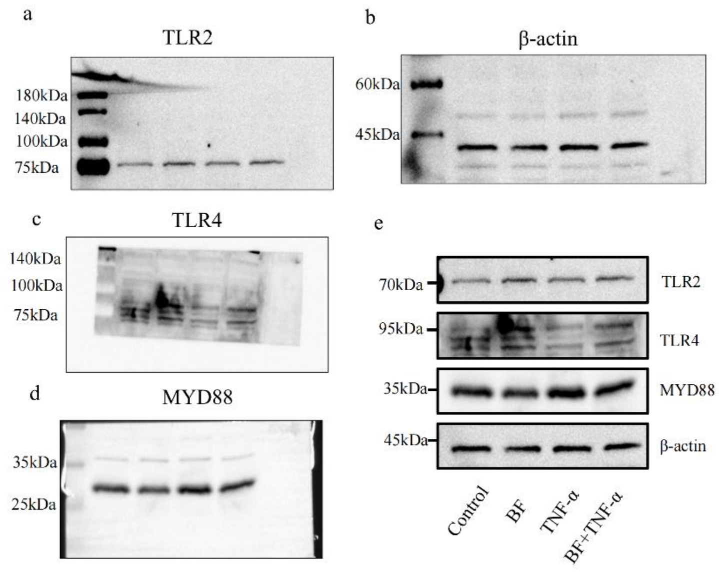

Protein bands in Figure5e.a-D: Original image of TLR2, TLR4, MYD88, β-actin.
f: Crop diagram of TLR2, TLR4, MYD88, β-actin.
