# Supplementary material for: Protective effects of a new generation of probiotic Bacteroides fragilis against colitis in vivo and in vitro
Source: Sci Rep. 2023 Sep 22;13:15842. doi: 10.1038/s41598-023-42481-8 (PMC10517118; doi:10.1038/s41598-023-42481-8)
Supplement: Supplementary file 2 — Supplementary Information 2. [file 41598_2023_42481_MOESM2_ESM.docx]

Additional file 2. The original western blot image was visible in Supplementary Material 2.
